# Heparin-binding protein as an emerging host-response biomarker for periprosthetic joint infection: a narrative review

**DOI:** 10.3389/fcimb.2026.1879154

**Published:** 2026-09-11

**Authors:** Qirui Chen, Khalid Waleed, Ai Peng, Shenghao Cai, Yong Xia, Zuorui Zheng, Khalid Waqar, Ammara Arooj, Jiang Shan, Zhou Junwei, Jianjian Deng, Bin Zhou, Xiaoling Fu

**Affiliations:** 1 Department of Orthopedics, The Second Affiliated Hospital of Nanchang University, Nanchang, China; 2 Jiangxi Medical College, Nanchang University, Nanchang, China; 3 Collaboration Unit for the State Key Laboratory of Infectious Disease Prevention and Control, Center for Evidence-Based Medicine, Jiangxi Provincial Key Laboratory of Disease Prevention and Public Health, School of Public Health, Jiangxi Medical College, Nanchang University, Nanchang, China; 4 Department of Cardiology, Punjab Institute of Cardiology, Lahore, Pakistan; 5 University College of Pharmacy, University of the Punjab, Lahore, Pakistan

**Keywords:** biomarker, heparin-binding protein (HBP), joint infection, periprosthetic joint infection (PJI), synovial fluid

## Abstract

**Background:**

Joint infections, including periprosthetic joint infection (PJI), remain difficult to diagnose because conventional markers such as C-reactive protein, erythrocyte sedimentation rate, white blood cell count, and procalcitonin may lack specificity, particularly after surgery or in inflammatory conditions. Heparin-binding protein (HBP), also known as azurocidin 1, is a neutrophil-derived mediator that is rapidly released during bacterial infection and may provide additional diagnostic value.

**Methods:**

A narrative review was conducted using major English- and Chinese-language databases to identify clinical studies evaluating HBP in PJI, musculoskeletal infection, systemic infection, or infection-versus-inflammation settings. Studies reporting diagnostic performance measures, including area under the receiver operating characteristic curve, sensitivity, specificity, or cutoff values, were included.

**Results:**

HBP concentrations were generally higher in infected patients than in non-infected or inflammatory controls. Across included studies, reported HBP AUC values ranged from 0.693 to 0.968. In PJI, serum HBP showed AUCs of 0.856 and 0.968 in available studies, whereas synovial fluid HBP showed more modest individual accuracy. HBP also demonstrated value in differentiating bacterial infection from sterile inflammatory disease, including rheumatoid arthritis with superimposed bacterial infection.

**Conclusion:**

HBP is a biologically plausible adjunctive biomarker for the diagnosis of bacterial infection and PJI. However, current evidence remains heterogeneous, and further large-scale prospective studies are needed to standardize cutoff values and clarify its role in diagnostic algorithms.

## 1 Introduction

Heparin-binding protein (HBP), also known as azurocidin or cationic antimicrobial protein-37 (CAP37), is stored in neutrophil granules and rapidly released in response to bacterial stimuli (Bentzer et al., 2016; Fisher and Linder, 2017; Fisher et al., 2022). It enhances vascular permeability, activates leukocytes, and augments inflammatory responses, thereby affording innate immunity (Bentzer et al., 2016; Fisher and Linder, 2017). Unlike conventional acute-phase proteins such as C-reactive protein (CRP), which depend on hepatic synthesis and rise more slowly, HBP is released almost immediately after neutrophil activation. HBP is a biologically plausible early biomarker of bacterial infection due to its rapid kinetic profile (Yang et al., 2019; Fisher et al., 2022).

Septic arthritis and periprosthetic joint infection (PJI) are serious orthopedic infections that result in significant morbidity, functional disability, and health care costs. Septic arthritis is associated with rapid cartilage destruction, and PJI is a leading cause of prosthesis failure and revision surgery (Trampuz and Zimmerli, 2007; Wu et al., 2014; Parvizi et al., 2018). Early diagnosis must therefore be the key to solving the problem, which is not simple. Acute postoperative PJI can have non-specific symptoms, and chronic or low-grade infection can be confused for an aseptic loosening, causing a delay in diagnosis (Trampuz and Zimmerli, 2007; Parvizi et al., 2013).

Diagnosis is currently based on clinical findings, microbiological cultures, and inflammatory markers, including procalcitonin (PCT), white blood cell count (WBC), erythrocyte sedimentation rate (ESR), and C-reactive protein (CRP). But each has its restrictions. Culture is labor-intensive and may be misleadingly negative when antibiotic use has occurred previously (Trampuz and Zimmerli, 2007; Parvizi et al., 2013). CRP and ESR lack specificity, as they can be elevated after surgery and in sterile inflammatory conditions (Ghanem et al., 2009; Tian et al., 2021). In localized joint infection, PCT sensitivity may be limited, but it is useful for systemic infection (Hemmann et al., 2024). Diagnostic uncertainty is even higher in patients with inflammatory or autoimmune conditions, who can have elevated CRP, ESR, and WBC levels without infection (Tian et al., 2021; Song et al., 2022). Similarly, postoperative inflammation can cause such trouble, so more specific and timely markers are needed (Ghanem et al., 2009).

Interest in HBP has grown due to its rapid release and its close association with neutrophil activation in bacterial infections. HBP has been reported to perform well in the diagnosis of sepsis and bacteremia, with areas under the curve (AUCs) above 0.88 and superior to several traditional markers in some cohorts (Taha et al., 2024; Mao et al., 2025). It could also be used to differentiate bacterial infection from sterile inflammatory activity: increased HBP levels in patients with superimposed bacterial infection and in those with noninfectious inflammatory flares, with reported AUCs up to 0.95 (Tian et al., 2021; Song et al., 2022).

Recent orthopedic evidence suggests that HBP may be beneficial in musculoskeletal infections. HBP shows promising diagnostic utility in orthopedic and trauma patients compared with CRP, WBC, and PCT (Hemmann et al., 2024). Additional investigation of PJI also indicates diagnostic possibilities, but these results depend on the stage of the infection, the assay method used, and the type of sample (Yang et al., 2023; Guo et al., 2025; Zhou et al., 2025). Neutrophil-driven inflammation may be less prominent in some chronic or low-grade infections, leading to reduced performance.

Conclusively, HBP appears to be a potential, but yet unproven, joint infection biomarker. The importance of biological relevance and the need for further evaluation for its rapid release, but the lack of standardization in existing cut-off values, the heterogeneity of existing studies, and the limited prospective validation are important barriers for clinical implementation. Hence, this review summarizes the available diagnostic evidence for HBP in musculoskeletal infection, with particular emphasis on PJI, discusses its reported performance alongside established biomarkers, and explores its potential role in future diagnostic pathways. Previous reviews and meta-analyses have mainly focused on HBP in sepsis and systemic infection (Taha et al., 2024), whereas the present review focuses on its emerging diagnostic role in orthopedic infection and PJI, while using systemic infection studies as supporting biological and clinical context.

## 2 Methods

### 2.1 Study design and scope

A narrative review was conducted to evaluate the role of heparin-binding protein (HBP) in musculoskeletal infections, with particular emphasis on periprosthetic joint infection (PJI) and infection-versus-inflammation diagnostic contexts. The review was guided by the Scale for the Assessment of Narrative Review Articles (SANRA) (Baethge et al., 2019). As this was a narrative review, no protocol was registered, and no formal risk-of-bias assessment was performed.

### 2.2 Literature search strategy

A structured literature search was performed in the electronic databases of PubMed, Web of Science, Baidu Scholar, and Google Scholar to identify relevant studies published from January 2005 to December 2025. Both subject headings and keywords were used in the search strategy, along with the Boolean operators “AND” and “OR” to maximize the number of relevant studies retrieved. PubMed, Web of Science were used as the main databases, while Google Scholar and Baidu Scholar were used as supplementary sources to identify potentially relevant English- and Chinese-language studies that might not have been retrieved through the main databases. In addition, the bibliographies of selected articles were also searched for additional studies. The detailed search strategies and search terms used for each database are provided in **Supplementary Table 1**.

### 2.3 Selection and extraction

Studies included that: (1) included human participants; (2) measured HBP as a diagnostic biomarker in PJI, musculoskeletal infection, systemic bacterial infection, or infection-versus-inflammation settings and (3) provided a diagnostic performance measure (sensitivity, specificity, ROC curve area under the curve [AUC], or cutoff value). Reviews, case reports, editorials, conference abstracts without full text, animal studies, and *in vitro* studies were not included. Studies without an accessible full text were excluded to ensure complete data extraction.

The data retrieved from the studies comprised study design, population, sample (serum, plasma, or synovial fluid), assay methodology, assay-specific cut-off, and the diagnostic test performance parameters. The diversity of study designs and outcomes precluded meta-analyses; instead, a narrative synthesis was performed.

### 2.4 Study selection

After screening the retrieved literature, nine clinical studies that directly evaluated HBP were included in the primary narrative synthesis. These studies assessed HBP in different clinical settings, including PJI, orthopedic and trauma-related infection, systemic bacterial infection, and infection-versus-inflammation conditions. Three additional studies evaluating other PJI biomarkers were included only as contextual evidence for comparison and were not counted as primary HBP studies.

## 3 Results

### 3.1 Study characteristics

The studies included nine clinical studies directly evaluating HBP, which were included in the primary narrative synthesis. These studies included retrospective and prospective observational designs and covered different clinical populations, including patients undergoing revision arthroplasty, patients with suspected PJI, orthopedic and trauma patients with infection, and patients with systemic or inflammatory conditions in whom bacterial infection needed to be distinguished from non-infectious inflammation. Three additional studies evaluating other PJI biomarkers were included only to provide contextual comparison with HBP and were considered separately from the primary HBP evidence.

The types of samples tested for HBP included serum, plasma, and synovial fluid. Several studies specifically examined HBP in orthopedic populations, including patients undergoing total or revision joint arthroplasty. In contrast, others assessed HBP in diverse populations, including those with trauma, soft-tissue infections, or systemic infections like sepsis. Furthermore, several studies have evaluated HBP in inflammatory conditions to assess its potential to distinguish infection from sterile inflammatory processes.

**Table 1** summarizes the nine clinical studies that directly evaluated HBP across PJI, orthopedic infection, systemic infection, and infection-versus-inflammation settings. These studies form the main clinical evidence base of this review. Studies evaluating other PJI biomarkers were considered separately for contextual comparison.

**Table 1:** Representative and clinically relevant studies evaluating HBP in joint infections and related conditions.

Study (Author, Year)	Population & design	Sample type	HBP assay method	Diagnostic/reference criteria	Key findings (HBP performance)	Comparison with other biomarkers	Methodological considerations
Direct PJI evidence							
Guo et al., 2025	132 revision arthroplasty patients (38 PJI, 94 aseptic); retrospective study	Plasma	Immunoturbidimetric method	2018 evidence-based PJI definition (Parvizi et al.)	HBP higher in PJI; AUC = 0.856 (95% CI 0.784–0.928); cutoff 51.3 ng/mL (sensitivity 69.2%, specificity 89.5%)	CRP, ESR, and WBC were elevated, but comparative AUCs were not reported	Retrospective, single-center PJI study; limited infected subgroup.
Zhou et al., 2025	135 revision arthroplasty patients (65 PJI, 70 aseptic failure); prospective diagnostic study	Serum	Commercial ELISA kit (Shanghai XuanKe Biotechnology); serum HBP	2014 MSIS criteria for PJI	HBP markedly higher in PJI; AUC = 0.968 (95% CI 0.943–0.993); cutoff 17.45 ng/mL (sensitivity 93.85%, specificity 81.58%)	Outperformed CRP (AUC 0.760), ESR (0.825), IL-6 (0.875), and PCT (0.663)	Prospective, single-center PJI study; selected revision population.
Yang et al., 2023	196 post-arthroplasty patients (36 PJI, 160 controls); retrospective study	Synovial fluid	Enzyme-linked immunosorbent assay (ELISA) for synovial-fluid HBP	Postoperative PJI diagnosed according to postoperative clinical status/diagnosis used by the study; exact named consensus criteria were not stated	Synovial fluid HBP was elevated in PJI; AUC = 0.693; cutoff = 14.43 ng/mL; sensitivity = 42.22%; specificity = 94.12%; combined-marker model AUC = 0.931	CRP AUC 0.697 and ESR AUC 0.692; combined testing improved accuracy	Retrospective PJI study; limited infected subgroup and synovial HBP assessment.
Other orthopedic infection evidence							
Hemmann et al., 2024	222 patients with PJI, trauma, infections, or controls; prospective study	Serum	ELISA; RayBio^®^ ELH-AZU1 kit for AZU1/HBP	Clinical infection categories including PJI/peri-implant infection; PJI definitions referenced MSIS/ICM/EBJIS frameworks	HBP elevated in infection; AUC = 0.790 (95% CI 0.721–0.859); sensitivity 73.4%, specificity 83.3%	Better overall balance than CRP (AUC 0.776), PCT (0.656), and WBC (0.641)	Prospective study; heterogeneous orthopedic/trauma population may influence HBP levels.
Supporting infection/infection-versus-inflammation evidence							
Mao et al., 2025	146 sepsis patients (57 bacteremia, 89 non-bacteremia); observational cohort	Plasma	Commercial immunofluorescence dry-quantitation assay, Joinstar Biomedical Technology	Sepsis defined by Sepsis-3; bacteremia defined by positive blood culture	HBP significantly higher in bacteremia; AUC = 0.880 (95% CI 0.820–0.940); cutoff 95.7 ng/mL (sensitivity 88.6%, specificity 68.1%)	Higher AUC than CRP (0.73), PCT (0.78), WBC (0.56), and IL-6 (0.59)	Observational sepsis cohort; bacteremia-based and not joint-specific.
Ma et al., 2022	87 patients with respiratory tract infection: 43 bacterial and 44 non-bacterial; retrospective diagnostic study	Plasma	ELISA using Jet-iStar 3000 dry immunofluorescence analyzer and Joinstar reagents	RTI criteria from Guidelines for Diagnosis and Treatment of Respiratory Diseases; bacterial group required positive bacterial culture	HBP differentiated bacterial from non-bacterial RTI; AUC = 0.785; cutoff 24.17 ng/mL; sensitivity 82.1%, specificity 77.1%	Higher AUC than PCT (0.767) and CRP (0.748); HBP + CRP improved AUC to 0.797	Retrospective respiratory-infection study; indirect evidence for orthopedic infection.
Song et al., 2022	Patients with rheumatoid arthritis with bacterial infection versus noninfectious flare	Serum	Immunoturbidimetric HBP assay	RA patients classified according to presence/absence of clinically diagnosed bacterial infection; not a PJI study	HBP showed strong discrimination of bacterial infection; AUC = 0.949 (95% CI 0.876–0.985); sensitivity 92.9%, specificity 84.6%	CRP and ESR had lower AUCs of approximately 0.80	RA infection-versus-inflammation study; not PJI-specific.
Tian et al., 2021	Patients with adult-onset Still’s disease versus sepsis	Serum	Commercial ELISA kit, Joinstar HBP20S1A02F, Jet-iStar 3000 analyzer	AOSD: Yamaguchi criteria; sepsis/septic shock: Sepsis-3 definitions	HBP differentiated sepsis from sterile inflammation; AUC up to 0.811	CRP and ESR were elevated in both groups	Sepsis-versus-AOSD study; indirect evidence for orthopedic infection.
Linder et al., 2012	179 critically ill ICU patients, including 151 with severe sepsis or septic shock and 28 non-septic controls; prospective study	Plasma	In-house ELISA using monoclonal anti-HBP antibody 2F23A	Sepsis/severe sepsis/septic shock defined according to contemporary consensus criteria; sepsis required infection plus SIRS	HBP is significantly higher in sepsis; HBP ≥15 ng/mL and/or HBP/WBC ratio >2 identified 87.2% of sepsis patients; elevated HBP is associated with increased 28-day mortality	HBP-based detection identified a greater proportion of sepsis patients than lactate >2.5 mmol/L (87.2% vs 64.9%)	Prospective ICU sepsis cohort; not joint-specific and no PJI-specific ROC analysis.

HBP, heparin-binding protein; PJI, periprosthetic joint infection; RA, rheumatoid arthritis; AUC, area under the curve; CRP, C-reactive protein; ESR, erythrocyte sedimentation rate; WBC, white blood cell count; PCT, procalcitonin.

Studies are grouped by relevance to PJI, including direct PJI evidence, other orthopedic infection evidence, and supporting infection/infection-versus-inflammation evidence. Methodological considerations are provided descriptively to aid interpretation of the reported diagnostic estimates and do not constitute a formal risk-of-bias or study-quality assessment. Consider differences in study populations, sample types, assay methods, and diagnostic criteria when comparing results across studies.

### 3.2 HBP in periprosthetic joint infection

Several studies have evaluated the diagnostic value of heparin-binding protein (HBP) in periprosthetic joint infection (PJI). Guo et al. reported that plasma HBP levels were significantly higher in patients with PJI than in those with aseptic failure, with an AUC of 0.856, a cutoff value of 51.3 ng/mL, sensitivity of 69.2%, and specificity of 89.5% (Guo et al., 2025). More recently, Zhou et al. prospectively evaluated serum HBP in 135 patients undergoing revision arthroplasty and found markedly higher HBP levels in patients with PJI than in those with aseptic failure. In that study, serum HBP showed an AUC of 0.968 (95% CI 0.943–0.993), a cutoff value of 17.45 ng/mL, sensitivity of 93.85%, and specificity of 81.58%, outperforming CRP, ESR, IL-6, and PCT in the same cohort (Zhou et al., 2025).

Synovial fluid HBP has also been investigated in postoperative PJI. Yang et al. retrospectively evaluated 196 elderly patients after joint replacement, including 36 with postoperative PJI and 160 without PJI. Synovial fluid HBP was significantly higher in infected patients and demonstrated an AUC of 0.693, with a cutoff value of 14.43 ng/mL, sensitivity of 42.22%, and specificity of 94.12%. Although the individual diagnostic performance of synovial HBP was modest, combining HBP with CRP, ESR, and miR-146a improved the AUC to 0.931 (Yang et al., 2023).

Across the available PJI studies, HBP concentrations were consistently higher in infected patients than in controls. Reported AUC values ranged from 0.693 to 0.968 across serum and synovial fluid studies. However, these findings were obtained from different cohorts and study designs, and therefore do not allow a direct comparison between serum and synovial fluid HBP. A comparison of the diagnostic findings is shown in **Table 1**.

### 3.3 HBP in differentiating bacterial infection from sterile inflammation

Additional studies have examined the value of heparin-binding protein (HBP) in distinguishing bacterial infection from non-infectious inflammatory activity. In a study of 122 patients with rheumatoid arthritis, those with coexisting bacterial infection had significantly higher plasma HBP concentrations than those with rheumatoid arthritis flares or in remission. HBP levels fell in response to antibiotics.

Receiver operating characteristic curve (ROC) analysis in this cohort showed an area under the curve (AUC) of 0.949 for HBP to discriminate between infection and non-infectious disease activity. HBP at a cut-off level of 24.55 ng/mL (approximately) demonstrated a sensitivity of 92.9% and specificity of 84.6%.

Other studies assessing HBP in inflammatory and infectious diseases have shown similar results, with higher HBP levels in patients with bacterial infections than in those with non-infectious inflammatory processes. In these studies, the reported AUCs ranged from 0.81 to 0.95, depending on the population studied and clinical circumstances.

In these studies, HBP concentrations were higher in patients with bacterial infection than in those with non-infectious inflammatory states, and decreased after successful treatment. **Table 1** summarizes the diagnostic performance of HBP in these infection-versus-inflammation settings.

### 3.4 Diagnostic performance of HBP vs other biomarkers

The diagnostic accuracy of heparin-binding protein (HBP) was assessed and compared with that of traditional inflammatory markers, including C-reactive protein (CRP), erythrocyte sedimentation rate (ESR), white blood cell count (WBC), and procalcitonin (PCT) across various cohorts.

In systemic infection, HBP had an area under the curve (AUC) of 0.88 for diagnosing bacteremia, whereas CRP, PCT, and WBC had AUCs of 0.73, 0.78, and 0.56, respectively.

In patients with orthopedic infection, HBP (AZU1) had an AUC of 0.790, a sensitivity of 73.4%, and a specificity of 83.3%. In this cohort, CRP had an AUC of 0.776, while PCT and WBC had AUC values of 0.656 and 0.641, respectively.

A more recent prospective PJI study reported even stronger performance for serum HBP, with an AUC of 0.968, sensitivity of 93.85%, and specificity of 81.58%, outperforming CRP, ESR, IL-6, and PCT in the same cohort. The diagnostic accuracy of other biomarkers in cohorts with PJI was as follows: β-defensin (AUC 0.948, sensitivity 96.6%, specificity 83.0%). S100A8 (calgranulin A) had the highest diagnostic performance, with an AUC of 0.964, sensitivity of 97.4%, and specificity of 86.0%, followed by S100A9 (calgranulin B) with an AUC of 0.902, and α-defensin (HNP1-3) with an AUC of 0.933.

HBP had an AUC of 0.949, a sensitivity of 92.9%, and a specificity of 84.6% in patients with rheumatoid arthritis and bacterial infection. Whereas CRP and ESR had lower AUCs of 0.761 and 0.719, respectively.

Across the studies reviewed, HBP showed variable diagnostic performance, with reported AUCs ranging from 0.693 to 0.968, depending on the clinical setting, sample type, and infection phenotype. The reported diagnostic performance of HBP and other biomarkers is summarized in **Table 2**. Because these findings were obtained from different study populations, clinical settings, and assay methods, values from separate studies should not be interpreted as direct head-to-head comparisons. Direct comparisons are considered only when biomarkers were evaluated within the same study cohort.

**Table 2:** Reported diagnostic performance of HBP and other biomarkers across included studies.

Study	Clinical setting	Biomarker	AUC (95% CI)	Sensitivity (%)	Specificity (%)
A. Studies with direct within-cohort HBP and biomarker comparisons					
Mao et al., 2025	Sepsis (bacteremia)	HBP (AZU1)	0.880 (0.820–0.940)	88.6	68.1
		CRP	0.730 (0.640–0.830)	—	—
		PCT	0.780 (0.690–0.860)	—	—
		WBC	0.560 (0.450–0.670)	—	—
Hemmann et al., 2024	Orthopedic infection	HBP (AZU1)	0.790 (0.721–0.859)	73.4	83.3
		CRP	0.776 (0.702–0.850)	—	—
		PCT	0.656 (0.574–0.738)	—	—
Yang et al., 2023	Postoperative PJI in elderly patients after joint replacement	HBP (synovial fluid)	0.693 (NR)	42.22	94.12
		CRP	0.697 (NR)	88.91	46.31
		ESR	0.692 (NR)	44.43	91.23
		miR-146a	0.786 (NR)	63.92	82.03
		HBP + CRP + ESR + miR-146a	0.931 (NR)	83.32	91.95
Ma et al., 2022	Bacterial vs non-bacterial respiratory tract infection	HBP (plasma)	0.785 (NR)	82.1	77.1
		PCT	0.767 (NR)	77.3	79.1
		CRP	0.748 (NR)	83.9	69.6
		HBP + CRP	0.797 (NR)	80.9	80.0
Zhou et al., 2025	PJI	HBP (serum)	0.968 (0.943–0.993)	93.85	81.58
		CRP	0.760 (0.680–0.840)	81.52	64.29
		ESR	0.825 (0.753–0.896)	83.08	77.14
		IL-6	0.875 (0.816–0.935)	87.69	84.29
		PCT	0.663 (0.567–0.759)	76.92	62.86
B. Additional HBP studies					
Guo et al., 2025	PJI	HBP (AZU1) (plasma)	0.856 (95% CI 0.784–0.928)	69.2	89.5
Song et al., 2022	RA + infection	HBP (AZU1)	0.949 (0.876–0.985)	92.9	84.6
C. Contextual PJI biomarker evidence (indirect comparison)					
Fernández-Torres et al., 2024	PJI	β-defensin	0.948 (0.909–0.987)	96.6	83.0
		CRP	0.902 (0.842–0.962)	93.0	77.1
		ESR	0.884 (0.811–0.956)	88.7	79.1
		WBC	0.767 (0.670–0.864)	82.0	68.2
Xu et al., 2023	PJI	Calgranulin A (S100A8)	0.964 (0.929–0.998)	97.4	86.0
		Calgranulin B (S100A9)	0.902 (0.823–0.980)	87.2	88.4
		α-defensin (HNP1–3)	0.933 (0.884–0.982)	89.7	83.7
Qin et al., 2020	PJI	ESR	0.719 (0.693–0.847)	63.64	70.15
		CRP	0.761 (0.675–0.835)	81.13	65.67

HBP, heparin-binding protein; AZU1, azurocidin 1; PJI, periprosthetic joint infection; RA, rheumatoid arthritis; AUC, area under the curve; CRP, C-reactive protein; ESR, erythrocyte sedimentation rate; WBC, white blood cell count; PCT, procalcitonin; IL-6, interleukin-6; S100A8, calgranulin A; S100A9, calgranulin B; HNP1–3, human neutrophil peptides 1–3 (α-defensin);.

AUC values and 95% CIs are reported as provided in the original studies; NR indicates not reported. Studies in Section A provide within-cohort HBP comparisons, whereas Section C presents contextual PJI biomarker evidence only. Comparisons across different studies are indirect because of differences in populations, assays, study designs, and diagnostic criteria.

## 4 Discussion

### 4.1 Principal findings

This review aims to present a comprehensive overview of the current evidence on the diagnostic value of heparin-binding protein (HBP) in joint infections and related clinical situations. The diagnostic accuracy of HBP ranged from 0.693 to 0.968 across various clinical scenarios and samples. In systemic infections, HBP showed an AUC of 0.880 for bacteremia, which was better than several traditional markers, including C-reactive protein (CRP), procalcitonin (PCT), and white blood cell (WBC) count (Mao et al., 2025). Likewise, in orthopedic and trauma infections, HBP (or azurocidin 1, AZU1) had an AUC of 0.790 and moderate sensitivity and specificity, offering potential for a wide range of clinical applications (Hemmann et al., 2024). For periprosthetic joint infection (PJI), reported HBP performance varied across studies. The reported AUCs for serum HBP in the available studies were 0.856 and 0.968. Synovial fluid HBP has also been evaluated, but the available evidence is limited and comes from separate cohorts with different study designs (Guo et al., 2025; Zhou et al., 2025). Furthermore, HBP showed an AUC of 0.949 in differentiating bacterial infection from non-infectious inflammatory disorders (such as rheumatoid arthritis) (Song et al., 2022). Despite this, other novel biomarkers, such as S100A8/S100A9 (calgranulin A/B), α-defensin, and β-defensin, have also shown high reported AUC values in separate PJI cohorts (Xu et al., 2023; Fernández-Torres et al., 2024). Overall, the available data suggest that HBP may have diagnostic value in bacterial infection across several clinical settings. But the heterogeneity in diagnostic performance and the availability of other high-performing biomarkers call for cautious interpretation and consideration of the clinical context.

### 4.2 Understanding the role of heparin-binding protein in diagnosis

Heparin-binding protein (HBP) or azurocidin 1 (AZU1) is a multifunctional protein that is, in part, secreted by activated neutrophils and plays an important role in the early immune reactions to bacterial infection. The main difference between HBP and conventional inflammatory markers is that HBP is a preformed and stored in the secretory vesicles and azurophilic granules of neutrophils, and is thus released immediately in response to bacterial stimulation, including endotoxins and pathogen-associated molecular patterns (PAMPs) (Linder et al., 2009; Bentzer et al., 2016).

Neutrophils release HBP into the extracellular environment and systemic circulation upon activation, where it exerts multiple effects involved in the inflammatory process. It is an important mediator of endothelial cell permeability. The effects of HBP on endothelial cells include cytoskeletal remodeling and contraction, leading to increased vascular permeability and increased tissue swelling, typical of acute bacterial infections (Linder et al., 2012). Furthermore, HBP stimulates the recruitment of leukocytes to the site by upregulating adhesion molecules and chemotactic factors, thereby increasing the inflammatory response (Fisher et al., 2022).

These characteristics distinguish HBP from traditional inflammatory and infectious biomarkers such as C-reactive protein (CRP) and procalcitonin (PCT), which are *de novo* synthesized and tend to rise late in the course of inflammation or infection. Unlike the slow-release profile of other circulating biomarkers, HBP can be detected early in infection, before the elevation of these other biomarkers, and thus has a rapid-release profile (Yang et al., 2019).

**Figure 1:**
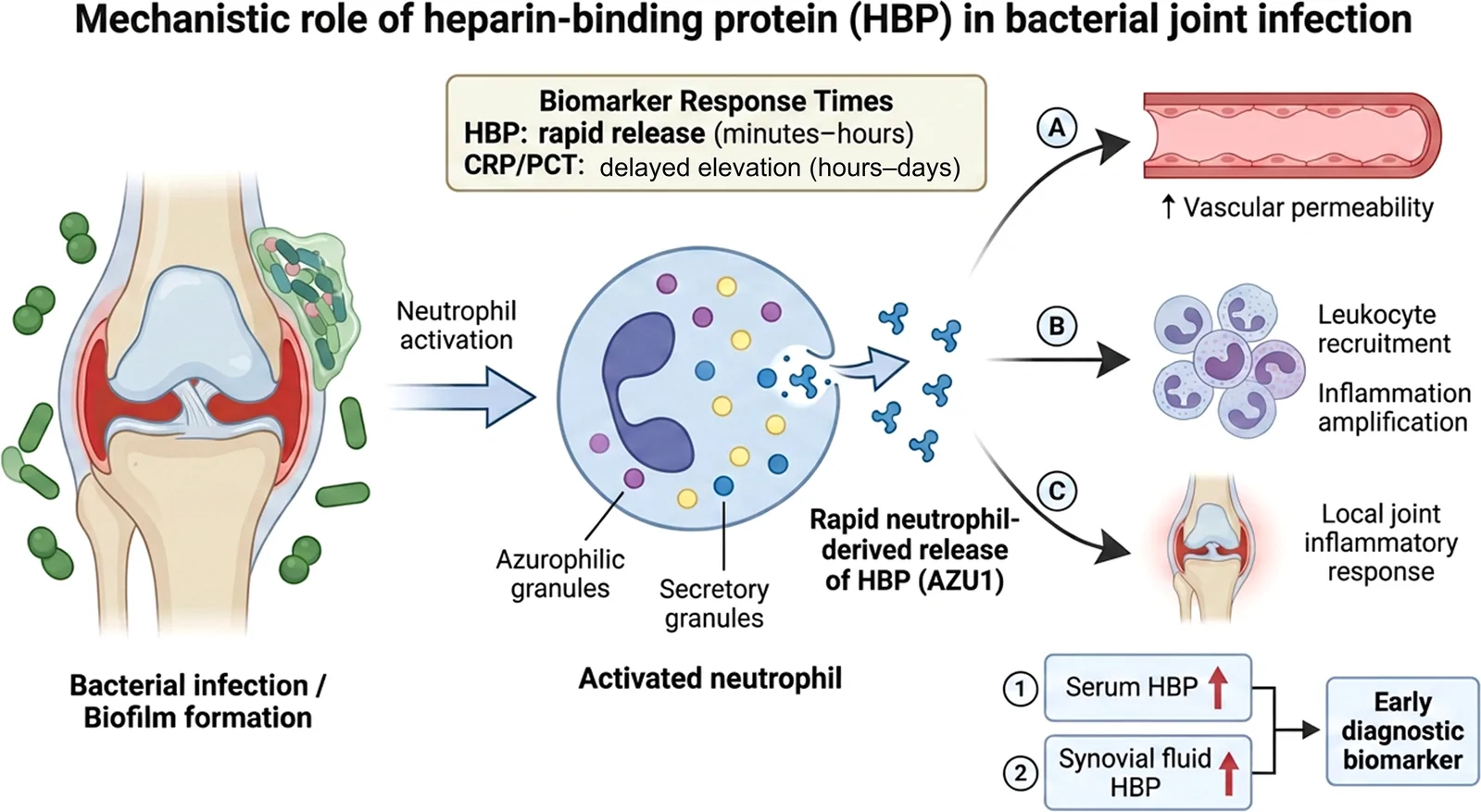
Mechanistic role of heparin-binding protein (HBP) in bacterial joint infection. Mechanistic role of heparin-binding protein (HBP) in bacterial joint infection. Neutrophil activation induces the rapid release of HBP (AZU1), thereby promoting endothelial leakage, amplifying inflammation, and increasing HBP levels in serum and synovial fluid, facilitating early detection of infection.

This enables HBP to be very sensitive to acute and systemic infections. Infection and inflammation are shown in **Figure 1** to be regulated by HBP. When bacteria invade, neutrophils are activated and release HBP immediately, causing endothelial damage, making the vessels leaky, and activating inflammatory cascades. This increases the concentration of HBP in blood and synovial fluid, and is useful as a diagnostic marker.

To conclude, the biological characteristics of HBP, including its immediate release, direct involvement in endothelial dysfunction, and propagation of inflammatory pathways, explain its usefulness in the diagnosis of various infectious diseases. The possible mechanism of HBP release and the biological consequences in bacterial joint infection are shown in **Figure 1**.

### 4.3 Performance of HBP in joint infections

Heparin-binding protein (HBP) is a potentially useful marker for the diagnosis of joint infections, especially periprosthetic joint infection (PJI); however, studies on its utility have yielded divergent results depending on the sample type, infection phenotype, and patient population.

Serum HBP had acceptable diagnostic performance in PJI. One study had an AUC of 0.856, with a sensitivity of 69.2% and a specificity of 89.5%. In contrast, another recent one was prospective and had an AUC of 0.968, a sensitivity of 93.85%, and a specificity of 81.58% (Guo et al., 2025; Zhou et al., 2025). These findings indicate that serum HBP could be beneficial for differentiating infection from aseptic failure in revision surgery. In addition, synovial fluid HBP has been evaluated, although the available evidence remains limited. In the study by Yang et al., the synovial fluid HBP had an AUC of 0.693, with a sensitivity of 42.22% and a specificity of 94.12%, but this was greatly enhanced when combined with CRP, ESR, and miR-146a (Yang et al., 2023).

The diagnostic value of HBP may vary by phase of infection. In acute infections with high neutrophil activation, HBP levels are elevated and rise rapidly, consistent with HBP serving as a biomarker of acute infections. Chronic or low-grade infections, on the other hand, can elicit a milder neutrophil-mediated response, potentially reducing diagnostic sensitivity in certain scenarios. HBP has also been useful in differentiating bacterial infection from sterile inflammatory activity, beyond PJI. In patients with RA, HBP levels were significantly elevated in those with a superimposed bacterial infection compared with those with non-infectious active disease, with an AUC of 0.949, a sensitivity of 92.9%, and a specificity of 84.6% (Song et al., 2022). This discovery indicates that HBP could be especially valuable in highly complex clinical scenarios where traditional inflammatory markers lack specificity.

To conclude, the diagnostic value of HBP in joint infection is promising for PJI, particularly in serum, and for differentiating infectious from non-infectious inflammation. However, the variation across studies suggests that its reported performance depends on clinical context, sample type, and stage of infection.

### 4.4 Comparison with other biomarkers

The diagnostic accuracy of heparin-binding protein (HBP) has recently been compared with that of other traditional and novel biomarkers for assessing joint infections. The reported diagnostic performance of HBP should be interpreted in relation to other available biomarkers.

HBP tends to perform as well as or better than conventional inflammatory biomarkers, including C-reactive protein (CRP), erythrocyte sedimentation rate (ESR), and white blood cell count (WBC) in acute and systemic infections (Linder et al., 2012; Wu et al., 2014; Yang et al., 2019). Traditional markers are routinely used in clinical settings; however, their diagnostic performance is often hindered by slower response kinetics and lack of specificity in inflammatory diseases (Trampuz and Zimmerli, 2007). In a recent prospective PJI study, serum HBP showed an AUC of 0.968, exceeding those of CRP, ESR, IL-6, and PCT measured in the same cohort, further supporting its potential value as an adjunctive serum biomarker for PJI diagnosis (Zhou et al., 2025).

Procalcitonin (PCT) is another widely used biomarker, which has demonstrated moderate diagnostic performance in musculoskeletal infections. But comparative studies suggest that HBP may be more sensitive in certain clinical situations, especially early in infection, although other studies have not shown differences between these biomarkers (Hemmann et al., 2024).

Several emerging biomarkers have also shown high reported AUC values in joint infections. S100A8 and S100A9 (calgranulin A/B) have demonstrated high diagnostic performance, with AUCs >0.90, indicating strong inflammatory activity via neutrophil activation. Likewise, α-defensin is well known for its high diagnostic accuracy for PJI, and β-defensin also shows high diagnostic accuracy in synovial fluid (Xu et al., 2023; Fernández-Torres et al., 2024). These biomarkers have also shown high diagnostic AUCs in separate PJI cohorts; however, these values should not be interpreted as evidence of superiority to HBP because they were obtained from different studies, especially in specific PJI populations (Xu et al., 2023; Fernández-Torres et al., 2024).

No single biomarker has demonstrated consistently superior diagnostic performance across all clinical settings. The inconsistency among biomarkers underscores the challenges of diagnosing joint infections, and using multiple markers may enhance diagnostic accuracy.

Overall, HBP may have value as an adjunctive biomarker because of its early release and association with neutrophil activity. But it is best used as an adjunct rather than a replacement, particularly when other highly sensitive markers are available.

### 4.5 Clinical implications

This study has identified several clinical implications for the diagnostic and therapeutic use of heparin-binding protein (HBP) in joint infections. HBP, released into the bloodstream within minutes of neutrophil activation, could be a useful early diagnostic marker for acute bacterial infections, for which early treatment is essential. Timely diagnosis is crucial in conditions like septic arthritis and periprosthetic joint infection (PJI), where a delay in diagnosis can result in joint damage, prosthesis failure, and greater morbidity (Trampuz and Zimmerli, 2007; Wu et al., 2014; Parvizi et al., 2018).

In the clinical setting, HBP could be beneficial when traditional biomarkers are inconclusive. Historically, markers such as C-reactive protein (CRP) and erythrocyte sedimentation rate (ESR) have been commonly used. Still, they may be non-specific, especially in the presence of inflammatory conditions or after surgery. In these cases, HBP may increase diagnostic certainty because neutrophils are involved in active infection (Ghanem et al., 2009; Tian et al., 2021).

Additionally, HBP’s ability to distinguish bacterial infection from non-infectious inflammation is valuable across diverse patient populations. For instance, in autoimmune diseases or chronic inflammatory conditions, it’s often difficult to differentiate between infection and disease flare. The increased HBP observed in infectious disease states suggests that it may be a valuable supplementary biomarker in these patients (Tian et al., 2021; Song et al., 2022).

Although HBP may be a promising diagnostic biomarker, it is unlikely to be used in isolation. Rather, its potential value may be in the context of multimarker approaches to diagnosis. The integration of HBP with traditional markers such as CRP and ESR, or with novel markers, may improve diagnostic performance and minimize misclassification (Ma et al., 2022; Yang et al., 2023; Zhou et al., 2025). Earlier and more accurate identification of bacterial joint infection may also support more timely treatment decisions and reduce unnecessary empirical antimicrobial exposure in cases where the diagnosis is uncertain.

In terms of its integration into clinical practice, the use of HBP will be influenced by factors such as test availability, cost considerations, and the implementation of standardized diagnostic thresholds. The absence of standardized criteria and conflicting reports currently hinders its integration. Thus, more large-scale, prospective studies are needed to determine the diagnostic criteria and ensure their use in clinical practice (Yang et al., 2023; Taha et al., 2024; Guo et al., 2025; Zhou et al., 2025).

In conclusion, HBP is a potential complementary marker for diagnosing joint infections, particularly as an early, biologically meaningful indicator of bacterial infection. The use of this protein in clinical practice, especially when combined with other biomarkers, may improve outcomes and aid diagnosis.

### 4.6 Limitations and future directions

There are several limitations of this review. First, the number of studies that specifically focus on the use of heparin-binding protein (HBP) in joint infections is relatively small, and most are conducted at a single center with small patient numbers. This may affect the reported strength of diagnostic accuracy and some of the discrepancies between studies (Yang et al., 2023; Hemmann et al., 2024; Guo et al., 2025; Zhou et al., 2025).

Secondly, there is great heterogeneity in the methods used, the patients enrolled, and the design. HBP performance may be influenced by variability in sample types (serum versus synovial fluid) collected, the assay methods used, and the different definitions of periprosthetic joint infection (PJI). In addition, the stage of infection (acute vs. chronic) could influence biomarker performance and make comparisons between studies difficult (Yang et al., 2023; Guo et al., 2025; Zhou et al., 2025).

Third, the lack of consistent HBP cut-off values limits its clinical application. The range of values of subjects excluded from analyses vary across studies because of differences in assays and study populations. This may make it difficult to establish a universally agreed-on diagnostic threshold and may affect diagnostic consistency.

Furthermore, although the HBP has potential as a diagnostic marker, there is a lack of comparative studies with other markers of interest. Other markers, such as α-defensin and S100 proteins, have been shown to have similar or better diagnostic accuracy. Still, systematic comparisons across large, well-characterized cohorts have not yet been conducted (Xu et al., 2023; Fernández-Torres et al., 2024).

This review also has methodological limitations, including the absence of formal risk-of-bias assessment and meta-analysis, exclusion of studies without accessible full text, and reliance on some indirect cross-study comparisons. These limitations should be considered when interpreting the findings.

Large, prospective, multicenter studies are needed to assess the diagnostic performance of HBP in various settings. The standardization of assay methods and diagnostic cut-off values will be important for the future use of this assay in clinical practice. In addition, studies comparing HBP with other biomarkers in combination could help determine the most effective diagnostic algorithm for joint infections. Investigations into its application for treatment response assessment and clinical outcome prediction can also increase its value.

## 5 Conclusion

Heparin-binding protein (HBP) is a biologically meaningful and highly responsive biomarker with potential for use in diagnosing joint infections. Its strong correlation with neutrophil activation and rapid release makes it a distinct marker compared with traditional inflammatory biomarkers, enabling early detection of bacterial infection across a variety of applications.

Available evidence suggests that HBP offers moderate to high diagnostic performance in PJI, systemic bacterial infection, and infection-versus-inflammation settings, while direct clinical evidence for HBP in septic arthritis is currently lacking. Although it offers performance comparable to (and, in some studies, superior to) established markers, heterogeneous results across studies and the availability of other strong-performing markers suggest that it should be interpreted with caution.

HBP is not intended to serve as a single diagnostic marker but rather as an adjunct to a multimarker diagnostic approach. Used in conjunction with traditional and new markers, HBP could enhance diagnostic accuracy, especially for complex or early infections.

Research should now focus on standardizing HBP assays, defining clinically relevant cut-offs, and conducting large prospective studies to better establish its role in diagnostic algorithms. With further validation, HBP may play a valuable role in enhancing diagnosis and patient care in joint infections.
