# Supplementary material for: Heparin-binding protein as an emerging host-response biomarker for periprosthetic joint infection: a narrative review
Source: Front Cell Infect Microbiol. 2026 Sep 11;16:1879154. doi: 10.3389/fcimb.2026.1879154 (PMC13613125; doi:10.3389/fcimb.2026.1879154)
Supplement: Supplementary file 1 [file Table1.docx]

**Supplementary Table S1. Detailed Literature Search Strategy**

**Search period:** January 2005 to December 2025. PubMed and Web of Science Core Collection were used as the primary databases. Google Scholar and Baidu Scholar were used as supplementary sources. The supplementary searches were targeted and the first 100 results ranked by relevance were screened for each query.

| **Source** | **Purpose** | **Full search strategy** | **Search yield / screening** |
| --- | --- | --- | --- |
| PubMed | Direct orthopedic/PJI evidence | (("heparin-binding protein"[Title/Abstract] OR "heparin binding protein"[Title/Abstract] OR HBP[Title/Abstract] OR azurocidin[Title/Abstract] OR AZU1[Title/Abstract]) AND ("periprosthetic joint infection"[Title/Abstract] OR "prosthetic joint infection"[Title/Abstract] OR PJI[Title/Abstract] OR "joint infection"[Title/Abstract] OR "orthopedic infection"[Title/Abstract] OR "orthopaedic infection"[Title/Abstract] OR "musculoskeletal infection"[Title/Abstract]) AND ("2005/01/01"[Date - Publication] : "2025/12/31"[Date - Publication])) | 3 records retrieved |
| PubMed | Supporting infection/inflammation evidence | (("heparin-binding protein"[Title/Abstract] OR HBP[Title/Abstract] OR azurocidin[Title/Abstract] OR AZU1[Title/Abstract]) AND ("rheumatoid arthritis"[Title/Abstract] OR "adult-onset Still's disease"[Title/Abstract] OR "infection versus inflammation"[Title/Abstract] OR "bacterial infection"[Title/Abstract]) AND ("2005/01/01"[Date - Publication] : "2025/12/31"[Date - Publication])) | 33 records retrieved |
| Web of Science Core Collection | Direct orthopedic/PJI evidence | TS=(("heparin-binding protein" OR "heparin binding protein" OR HBP OR azurocidin OR AZU1) AND ("periprosthetic joint infection" OR "prosthetic joint infection" OR PJI OR "joint infection" OR "orthopedic infection" OR "orthopaedic infection" OR "musculoskeletal infection")); publication years limited to 2005–2025. | 4 records retrieved |
| Web of Science Core Collection | Supporting infection/inflammation evidence | TS=(("heparin-binding protein" OR "heparin binding protein" OR HBP OR azurocidin OR AZU1) AND ("rheumatoid arthritis" OR "adult-onset Still's disease" OR "infection versus inflammation" OR "bacterial infection")); publication years limited to 2005–2025. | 40 records retrieved |
| Google Scholar | Direct orthopedic/PJI evidence | Exact phrase: "heparin-binding protein"; at least one of the words: periprosthetic, prosthetic, joint, orthopedic, orthopaedic, musculoskeletal; years 2005–2025. | Supplementary targeted search; first 100 relevance-ranked results screened |
| Google Scholar | Supporting infection/inflammation evidence | Exact phrase: "heparin-binding protein"; at least one of the words: rheumatoid, Still, bacterial, infection, inflammation; years 2005–2025. | Supplementary targeted search; first 100 relevance-ranked results screened |
| Baidu Scholar | Direct orthopedic/PJI evidence | Targeted Chinese searches: 肝素结合蛋白 假体周围关节感染; 肝素结合蛋白 关节感染; 肝素结合蛋白 骨科感染. | Supplementary targeted search; relevance-ranked results screened |
| Baidu Scholar | Supporting infection/inflammation evidence | Targeted Chinese searches: 肝素结合蛋白 类风湿关节炎 细菌感染; 肝素结合蛋白 成人斯蒂尔病 感染. | Supplementary targeted search; relevance-ranked results screened |

**Note:** The search was rerun during revision to improve transparency and reproducibility. The previous PRISMA-style numerical flow and the earlier 27-record yield were removed because the review was designed as a narrative review rather than a formal systematic review. Duplicate and clearly irrelevant records were excluded during screening, and studies were selected for the primary narrative synthesis according to their relevance to the diagnostic questions addressed in the review.
